# Sequence analysis of sickness absence and disability pension in the year before and the three years following a bicycle crash; a nationwide longitudinal cohort study of 6353 injured individuals

**DOI:** 10.1186/s12889-020-09788-x

**Published:** 2020-11-16

**Authors:** Linnea Kjeldgård, Helena Stigson, Kristina Alexanderson, Emilie Friberg

**Affiliations:** 1 Division of Insurance Medicine, Department of Clinical Neuroscience, Karolinska Institutet, SE-171 77 Stockholm, Sweden; 2 Vehicle Safety Division, Department of Applied Mechanics, Chalmers University of Technology, Gothenburg, Sweden; 3 Folksam Research, Folksam Insurance Group, Stockholm, Sweden

**Keywords:** Sick leave, Traffic injury, Sequence analysis, Longitudinal cohort, Bicycle crash, Disability pension, real-world data

## Abstract

**Background:**

Bicyclists are the road user group with the highest number of severe injuries in the EU, yet little is known about sickness absence (SA) and disability pension (DP) following such injuries.

**Aims:**

To explore long-term patterns of SA and DP among injured bicyclists, and to identify characteristics associated with the specific patterns.

**Methods:**

A longitudinal register-based study was conducted, including all 6353 individuals aged 18–59 years and living in Sweden in 2009, who in 2010 had incident in-patient or specialized out-patient healthcare after a bicycle crash. Information about sociodemographic factors, the injury, SA (SA spells > 14 days), and DP was obtained from nationwide registers. Weekly SA/DP states over 1 year before through 3 years after the crash date were used in sequence and cluster analyses. Multinomial logistic regression was used to estimate odds ratios (OR) and 95% confidence intervals (CI) for factors associated with each identified sequence cluster.

**Results:**

Seven clusters were identified: “No SA or DP” (58.2% of the cohort), “Low SA or DP” (7.4%), “Immediate SA” (20.3%), “Episodic SA” (5.9%), “Long-term SA” (1.7%), “Ongoing part-time DP” (1.7%), and “Ongoing full-time DP” (4.8%). Compared to the cluster “No SA or DP”, all other clusters had higher ORs for women, and higher age. All clusters but “Low SA and DP” had higher ORs for inpatient healthcare. The cluster “Immediate SA” had a higher OR for: fractures (OR 4.3; CI 3.5–5.2), dislocation (2.8; 2.0–3.9), sprains and strains (2.0; 1.5–2.7), and internal injuries (3.0; 1.3–6.7) compared with external injuries. The cluster “Episodic SA” had higher ORs for: traumatic brain injury, not concussion (4.2; 1.1–16.1), spine and back (4.5; 2.2–9.5), torso (2.5; 1.4–4.3), upper extremities (2.9; 1.9–4.5), and lower extremities (3.5; 2.2–5.5) compared with injuries to the head, face, and neck (not traumatic brain injuries). The cluster “Long-term SA” had higher ORs for collisions with motor vehicles (1.9;1.1–3.2) and traumatic brain injury, not concussion (18.4;2.2–155.2).

**Conclusion:**

Sequence analysis enabled exploration of the large heterogeneity of SA and DP following a bicycle crash. More knowledge is needed on how to prevent bicycle crashes and especially those crashes/injuries leading to long-term consequences.

## Background

Bicycling has a positive public health impact and increased bicycling is an important aspect of sustainable transportation [1, 2]. However, within the area of traffic safety, one of the largest challenges is the safety of bicyclists [1]. Today, bicyclists are the road-user group with the highest number of severe injuries in Sweden, as well as in all of the EU [3, 4]. Information on mileage data, number of bicyclists, and number of crashes including all injury severities are uncertain or non-existing at national levels. About 2000 bicyclists are severely injured in Sweden each year [5]. By using traffic mileage data, a Swedish study estimated a 29 times higher risk for injury among bicyclists compared with car occupants [6]. The majority of injuries are minor, but may still lead to long-term consequences, hence, a focus on nonfatal outcomes is essential. One such long-term consequence could be to what extent the injury impacts the function of the injured, in terms of her or his work capacity, indicated by sickness absence (SA), and disability pension (DP).

There is only limited scientific knowledge about SA and DP following a bicycle crash. Sickness absence has been shown to be a relatively common outcome after a road traffic injury [7–11]. However, there are only a few studies on bicyclists’ injuries and their future risk of SA [9, 12–15], and only one study considering this with a long-term perspective, in this case, the duration of the SA spell starting in connection to the bicycle crash [14]. Much has happened in the traffic situation since these older studies were conducted, e.g., the road traffic safety policy Vision Zero, adopted by the Swedish parliament in 1997 [16–18].

Sickness absence and DP has consequences not only for the individual, but also for the individuals’ family, colleagues, employer, insurer, and for the whole of society [19, 20]. Sociodemographic factors, such as educational level, country of birth, marital status, gender, age, as well as previous SA, are well known to be associated with SA and DP [20–25]. Many different measures of SA are used to capture the inherent complexity of it, with regard to recurrent spells, skewed distribution, different durations, and varying extent of the spells [26, 27]. Previously, mainly different types of traditional regressions analyses have been used in analyses of risks regarding SA and DP and other such events [25, 28]. Accordingly, the focus in those studies was either on a single timepoint in a cross-sectional study, or at the end of follow-up in a longitudinal study. Sequence analysis could be a suitable method to also gain knowledge on different patterns over time regarding, e.g., individuals’ timing, duration, and order of different types of events, such as SA and DP [29] and the interest for such analyses has increased lately. Sequence analysis has been used in several studies, with the observation that the identified heterogeneity between the sequences can be a good complement and adds additional value to traditional regression analyses [30–32]. Thus, to get a more complete picture of the long-term patterns regarding SA and DP in relation to a bicycle crash there is a need for studies using more comprehensive methods.

Therefore, *the aims* of this study were to explore and identify patterns of SA and DP among injured bicyclists and to find characteristics associated with the specific patterns identified.

## Methods

A population-based longitudinal cohort study was conducted, based on nationwide register microdata, to investigate patterns of SA and DP during the period of 1 year before through 3 years after the date of a bicycle crash. The study population included all individuals aged 18–59 years, living in Sweden 31 December 2009, who in 2010 received in-patient or specialized out-patient healthcare due to an injury from a new bicycle crash. The age limits were chosen to reflect eligibility of SA and DP during follow-up.

### Data sources

Data from five nationwide registers from the following three authorities were used and linked at the individual level, using the unique personal identity number assigned to all residents in Sweden [33]:

- From *Statistics Sweden*, the “Longitudinal integration database for health insurance and labour market studies” (LISA) was used to identify all individuals aged 18–59 years living in Sweden 31 December 2009, *N* = 5,096,121, and for sociodemographic information (sex, age, level of education, country of birth, type of living area, and marital status in December 2009 and for emigration in 2010–2013).
- Three registers from the *National Board of Health and Welfare:* The in-patient and specialized out-patient healthcare registers were used to identify those injured in a bicycle crash and for medical information related to the injury; and the cause of death register to identify those who died during the follow-up.
- From the *Swedish Social Insurance Agency*, the register “Micro-data for analyses of the social insurance” (MiDAS) was used for dates and grades of all SA spells > 14 days and all DP.

### The cohort

In the in-patient and specialized out-patient healthcare registers, both diagnoses (main and all secondary diagnoses) and external causes of morbidity are recorded according to the International Statistical Classification of Diseases and Related Health Problems; ICD-10 [34]. Included in the study were the individuals that received in-patient or specialized out-patient healthcare in 2010 due to bicycle crashes (identified by the ICD-10 codes for external causes of morbidity V10-V19: “Pedal cycle rider injured in transport accident”) among those that had an injury diagnosis as main or secondary diagnoses (ICD10: S00-T89 “Injury, poisoning and certain other consequences of external causes” or Z04.1 “Examination and observation following transport accident”). Excluded were those who during the 3 years before the date of this first in-patient or specialized out-patient healthcare due to a bicycle crash in 2010 had received in-patient or specialized out-patient healthcare for a bicycle or another transport-related injury (ICD-10 external causes of morbidity V00-V99: “Transport accidents”), leaving 6465 individuals. To ensure complete follow-up data, individuals who died or emigrated during the follow-up were also excluded (*n* = 112), resulting in 6353 individuals for inclusion in the study cohort. The date of the visit/hospitalization is here referred to as the *presumed crash date.* From these data sources, we do not know the actual date of the crash, it might have been a few days before they had the specialized healthcare visit, e.g., having first sought primary healthcare or waiting to seek healthcare for other reasons.

### Sickness absence and disability pension in Sweden

All individuals living in Sweden, ≥16 years old, and with income from work, unemployment, or parental-leave benefits can get SA benefits if having a disease or injury leading to reduced work capacity [35]. The first day of a SA spell is an unreimbursed qualifying day (varying number of days for self-employed). A physician’s certificate is required from the eighth day. For most employees, day 2–14 are reimbursed by the employer, thereafter, from the Social Insurance Agency. For others, e.g., unemployed, the Social Insurance Agency pays the benefits from the second SA day, thus, information also on shorter SA spells was available for those individuals. In this study, in order not to introduce a bias, only SA spells > 14 days were included. All individuals aged 19–64 can be granted DP if disease or injury leads to long-term or permanent work incapacity. Both SA and DP can be granted for full- or part-time (100, 75, 50, 25%) of ordinary work hours. That is, someone on part-time DP can at the same time have part-time SA.

### Definition of SA/DP states/week

States regarding SA and DP were defined weekly for each of the 6353 individuals, into five non overlapping states: No SA or DP (no SA or DP during the week); SA (any SA during the week, and no DP); SA and DP (any SA and any DP during the week); part-time DP (any part-time DP during the week and no SA and no full-time DP); and full-time DP (any full-time DP during the week and no SA). All SA and DP irrespective of the diagnoses were included in the analyses. Week zero (W_0_) was defined as the presumed crash date, the 3 days before that day and the 3 days after that day (that is, seven days). Many individuals in this cohort had already ongoing SA and DP in connection to the crash and several studies have shown associations between previous and future SA and DP [24, 36–39]. Therefore, to capture the full picture of SA and DP, all individuals were followed from 1 year before (W_−52_) through 3 years after (W_+156_) W_0_.

### Crash and injury characteristics

Based on type of crash, the individuals were categorized into the following three groups: single-bicycle crash (ICD-10: V17, V18, V19.3, V19.8, V19.9) (reference group); collision with pedestrian, animal, or other bicycle (V10, V11); and collision with motor vehicle (V12-V16, V19, V19.0, V19.1-V19.2, V19.4-V19.6). Single-bicycle crash consists of crashes such as bicycle rider injured in a collision with fixed or stationary object, non-collision transport accident (fall or thrown from bicycle), and unspecified traffic accident (of which the presumed majority are single). In-patient healthcare was categorized into two groups as: No (only visits to specialized out-patient healthcare at the presumed crash date) (reference group); and yes (in-patient healthcare at the presumed crash date, may also have a specialized out-patient healthcare visit during the same day).

Some individuals had up to three different healthcare visits registered in the patient registers on the presumed crash date, e.g., due to being transferred to another type of clinic. Each visit had a main diagnosis and could also have a number of additional secondary diagnoses. For categorization purposes, we selected one injury diagnosis per individual, in the following way: The main injury diagnosis was selected over secondary injury diagnoses, the diagnoses for in-patient healthcare over out-patient healthcare, and injury before other types of diagnoses (S00-S99 over T00-T88, T00-T88 over Z04.1). The majority (78%) had only one injury diagnosis, 15% had two, and 7% had more than two.

A modified version of the Barell matrix [8, 40] was used to classify the ICD-10 codes into categories of type of injury and into injured body region. Thus, *type of injury* was categorized into the following six groups: fracture; dislocation; sprains and strains; internal (including: brain, spinal cord, and other internal organs); external (including: open wounds, contusions and superficial injuries) (reference group); and other and unspecified. The *injured body region* was categorized into eight groups: ‘head, face, and neck, not traumatic brain injury (TBI)’ (reference group); ‘TBI, not concussion’; ‘concussion’; ‘vertebral column and spinal cord’; ‘torso’; ‘upper extremities’; ‘lower extremities’; and ‘other and unspecified’. Moreover, the three groups ‘head, face, and neck, not TBI’; ‘TBI, not concussion’; and ‘concussion’ were in some analyses collapsed into the group ‘all head and neck’.

### Sociodemographic factors

The sociodemographic factors were categorized as: sex (women; men (reference group)), age group (18–40 (reference group); 41–59 years), level of education (elementary (≤9 years and missing (missing = 109 individuals)); high school (9–12 years); university/college (> 12 years) (reference group)), country of birth (Sweden (reference group); not Sweden), type of living area (big cities (reference group); medium-sized cities; small cities/villages), marital status (married (reference group); not married). Reference groups were chosen based on size of the groups and expected proportions with new SA, with either larger groups or groups expected to have lower proportions of new SA being used as the reference.

### Statistical analysis

The individual’s sociodemographic factors (age, level of education, country of birth, type of living area, and marital status), crash type, type of healthcare, type of injury, and injured body region were calculated stratified by sex, using descriptive statistics. The descriptive analyses were stratified by sex, since women have higher risk for SA [21]. In the further analyses, to not lose statistical power, women/men were studied together and the proportion of each could be compared.

Sequences of SA/DP states/week were estimated during a four-year period (from 1 year before and through 3 years after W_0_ (W_−52_ to W_+156_)) with sequence analysis using TraMineR in R [41]. Thereafter, cluster analysis with optimal matching spell [42] were used to find different clusters of individuals who had similar sequences of SA/DP-states. A cluster tree and several measures of cluster partition quality were used to choose the number of clusters. Density plots for the clusters show the density of each state every week for all clusters. Representative sequences show the sequence(s) that with a neighbourhood radius of 10% cover(s) at least 25% of all sequences in each cluster.

Multinomial logistic regression models were used to analyse the association between sociodemographic factors, crash type, type of healthcare, type of injury, injured body region, and SA/DP-clusters. Crude and mutually adjusted odds ratios (OR) and 95% confidence intervals (CI) were calculated. Sensitivity analyses were conducted including the 112 individuals who died or emigrated during the follow-up. The statistical analyses were performed using R (version 3.5.0).

## Results

Of the 6353 individuals aged 18–59 years with incident in-patient or specialized out-patient healthcare due to a new bicycle crash in 2010, 57% were men (Table 1). Mean age of the cohort was 39 years and a majority had high school or university education (83%). The most frequent crash type was single bicycle-crashes (85%). External injuries and fractures were the most frequent injury types, 39 and 36% of the injuries, respectively. The most frequently injured body regions were ‘upper extremities’ (43%) followed by ‘all head and neck’ (28%).

**Table 1:** Sociodemographic and injury characteristics, by sex, among the study population^a^

	All	Women	Men
	n (%)	n (%)	n (%)
**Total (row%)**	6353 (100)	2729 (43.0)	3624 (57.0)
**Age group**^**b**^			
18–40 years	3122 (49.1)	1298 (47.6)	1824 (50.3)
41–59 years	3231 (50.9)	1431 (52.4)	1800 (49.7)
**Level of education**^**b**^			
Elementary	1078 (17.0)	391 (14.3)	687 (19.0)
High School	2908 (45.8)	1200 (44.0)	1708 (47.1)
University/College	2367 (37.3)	1138 (41.7)	1229 (33.9)
**Country of birth**^**b**^			
Sweden	5382 (84.7)	2290 (83.9)	3092 (85.3)
Not Sweden	971 (15.3)	439 (16.1)	532 (14.7)
**Type of living area**^**b**^			
Big cities	2229 (35.1)	965 (35.4)	1264 (34.9)
Medium-sized cities	2638 (41.5)	1152 (42.2)	1486 (41.0)
Small cities/villages	1486 (23.4)	612 (22.4)	874 (24.1)
**Marital status**^**b**^			
Married	2052 (32.3)	951 (34.8)	1101 (30.4)
Not married	4301 (67.7)	1778 (65.2)	2523 (69.6)
**Crash type**^**c**^			
Single	5378 (84.7)	2235 (81.9)	3143 (86.7)
Collision with pedestrian, animal, or other bicycle	366 (5.8)	188 (6.9)	178 (4.9)
Collision with motor vehicle	609 (9.6)	306 (11.2)	303 (8.4)
**Inpatient healthcare**^**c**^			
No	5304 (83.5)	2293 (84.0)	3011 (83.1)
Yes	1049 (16.5)	436 (16.0)	613 (16.9)
**Type of injury**^**c**^			
Fracture	2316 (36.5)	956 (35.0)	1360 (37.5)
Dislocation	280 (4.4)	74 (2.7)	206 (5.7)
Sprains and strains	566 (8.9)	266 (9.7)	300 (8.3)
Internal	604 (9.5)	262 (9.6)	342 (9.4)
External	2447 (38.5)	1108 (40.6)	1339 (36.9)
Other and unspecified	140 (2.2)	63 (2.3)	77 (2.1)
**Body region**^**c**^			
‘Head, face, and neck, not TBI’^d^	1224 (19.3)	495 (18.1)	729 (20.1)
‘TBI, not concussion’	79 (1.2)	28 (1.0)	51 (1.4)
‘Concussion’	484 (7.6)	222 (8.1)	262 (7.2)
*‘All head and neck’*^e^	*1787 (28.1)*	*745 (27.2)*	*1042 (28.7)*
‘Spine and back’	131 (2.1)	57 (2.1)	74 (2.0)
‘Torso’	458 (7.2)	192 (7.0)	266 (7.3)
‘Upper extremities’	2729 (43.0)	1052 (38.5)	1677 (46.3)
‘Lower extremities’	1158 (18.2)	647 (23.7)	511 (14.1)
‘Other and unspecified’	90 (1.4)	36 (1.3)	54 (1.5)

^a^6353 individuals aged 18–59 years injured in a bicycle crash in 2010 in Sweden

^b^31st of December 2009

^c^At the presumed crash date

^d^Abbreviation: *TBI* Traumatic Brain Injury

^e^Sum of the categories: ‘Head, face, and neck, not TBI’, ‘TBI, not concussion’, and ‘Concussion’

**Fig. 1:**
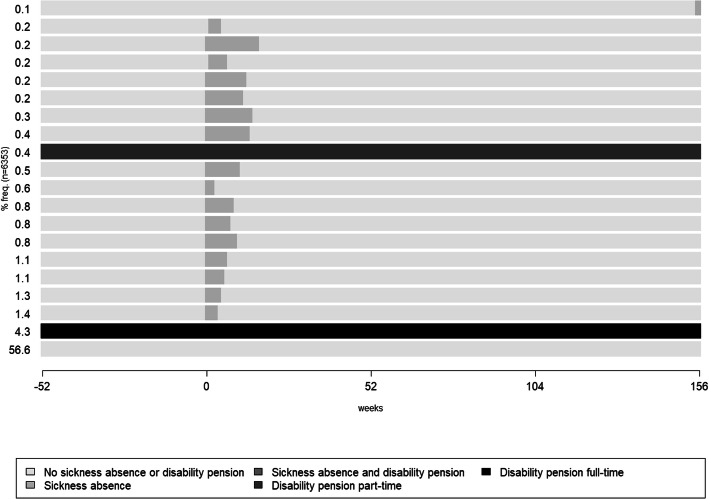
The 20 most common sequences of SA and/or DP states/week during one year before up until three years after (W_−52_ to W_+156_) the week of the bicycle crash (marked with 0 in the figure). The proportion of each sequence are stated at the y-axis

When first exploring all the sequences of SA and DP states/week among all 6353 individuals during the year before through 3 years after the week of the bicycle crash W_0_ (that is, W_−52_ to W_+156_) we found large variation in the patterns of SA and DP states/week among the individuals. The most frequently occurring sequence was having no SA or DP during all 4 years (W_−52_ to W_+156_) (56.6%), followed by the individuals with full-time DP during all 4 years (4.3%). Thereafter, a number of sequences with no SA or DP before the crash and with SA starting in connection to the crash with varying number of weeks of SA but after some time going back to the no SA or DP state for the rest of the follow-up. Part-time DP during the whole follow-up was the 12th most frequent sequence (0.4%) (Fig. 1).

**Fig. 2:**
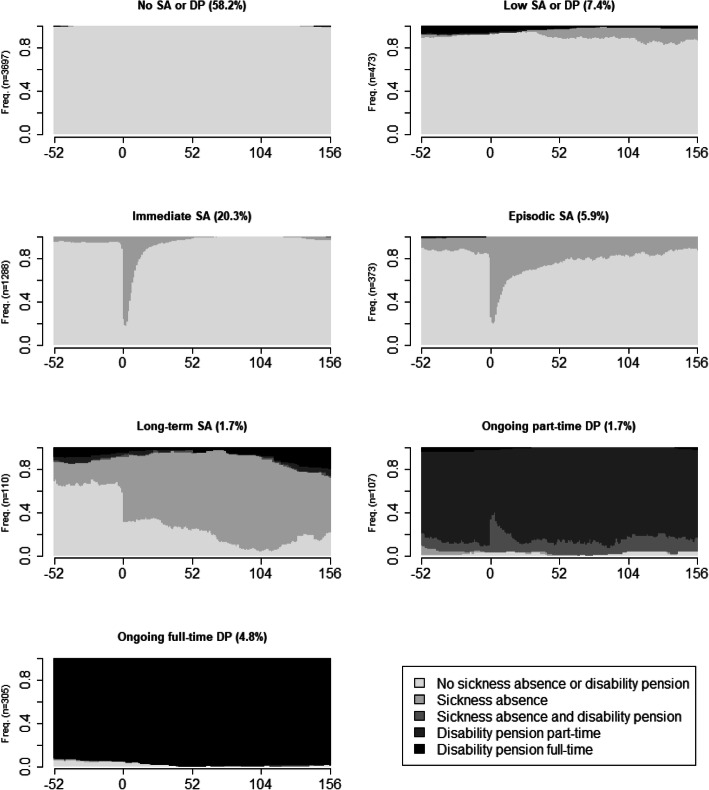
Density plots of sickness absence and/or disability pension states/week during the year before through three years after (W_−52_ to W_+156_) the week of the bicycle crash (marked with 0 in the figure), for seven identified clusters. The number of individuals in each cluster are given before the respective Y-axis and the proportion of the study population (*N* = 6353) is stated in each cluster heading

Thereafter, cluster analysis was used to identify clusters of sequences, and seven different clusters were identified. They were called: “No SA or DP” (including 58.2% of the cohort), “Low SA or DP” (7.4%), “Immediate SA” (20.3%), “Episodic SA” (5.9%), “Long-term SA” (1.7%), “Ongoing part-time DP” (1.7%), and “Ongoing full-time DP” (4.8%). The measures of cluster partition quality that were used to choose the number of clusters are presented in Table 2. In Fig. 2, the seven clusters are illustrated using density plots. Those in the “No SA or DP” cluster had almost no SA or DP during the 4 years (W_−52_ to W_+156_).

**Table 2:** Measures of cluster partitions quality for different number^a^ of clusters

	PBC^b^	HG^c^	HGSD^d^	ASW^e^	ASWw^f^	CH^g^	R2^h^	CHsq^i^	R2sq^j^	HC^k^
2 clusters	0.74	0.99	0.99	0.75	0.75	2063.58	0.25	4404.48	0.41	0.01
3 clusters	0.66	0.84	0.84	0.68	0.68	4232.97	0.57	5975.31	0.65	0.07
4 clusters	0.67	0.84	0.84	0.70	0.70	3589.46	0.63	6458.03	0.75	0.07
5 clusters	0.71	0.92	0.92	0.72	0.72	3419.08	0.68	6821.89	0.81	0.03
6 clusters	0.73	0.97	0.97	0.76	0.76	3502.94	0.73	6901.61	0.84	0.02
**7 clusters**	**0.73**	**0.98**	**0.98**	**0.77**	**0.77**	**3247.65**	**0.75**	**7341.25**	**0.87**	**0.01**
8 clusters	0.73	0.98	0.98	0.77	0.77	3039.62	0.77	7073.04	0.89	0.01
9 clusters	0.73	0.98	0.98	0.77	0.77	2786.74	0.78	6716.10	0.89	0.01
10 clusters	0.73	0.99	0.99	0.78	0.78	2587.42	0.79	6513.68	0.90	0.01
11 clusters	0.73	0.99	0.99	0.78	0.78	2410.38	0.79	6289.63	0.91	0.01
12 clusters	0.73	0.99	0.99	0.76	0.76	2324.17	0.80	6086.74	0.91	0.01
13 clusters	0.73	0.99	0.99	0.77	0.77	2270.38	0.81	5883.08	0.92	0.00

^a^The here selected number of clusters market in bold

^b^Point Biserial Correlation

^c^Hubert’s Gamma

^d^Hubert’s Somers’ D

^e^Average Silhouette Width

^f^Average Silhouette Width (weighted)

^g^Calinski-Harabasz index

^h^Pseudo R^2^

^i^Calinski-Harabasz index squared

^j^Pseudo R^2^ squared

^k^Hubert’s C

The characteristics of the clusters and the mutually adjusted associations for the different clusters compared to the cluster “No SA or DP” are shown in Table 3. The unadjusted ORs were not substantially altered when mutually adjusting for covariates accordingly, the unadjusted ORs are shown in Table 4. In the cluster “No SA or DP”, which also was the largest cluster, the most common type of injuries were external injuries (47%) and fractures (28%) and the most commonly injured body regions were ‘upper extremities’ (40%) followed by ‘head, face, and neck, not TBI’ (24%). Compared to this cluster, almost all other clusters where associated with a higher proportion of women, of individuals of older age group, and of individuals with high school education (compared with university/college). Further, all clusters but “Low SA and DP” had high ORs for inpatient healthcare at the presumed crash date. Proportions for which the crash resulted in inpatient healthcare was largest in the clusters “Long-term SA” (31%), “Episodic SA” (29%), and “Immediate SA” (28%) and smallest in the cluster “Low SA or DP” (10%).

**Table 3:** Distributions and mutually adjusted OR for the seven clusters of SA and DP

	No SA or DP		Low SA or DP			Immediate SA			Episodic SA			Long-term SA			Ongoing part-time DP			Ongoing full-time DP		
	n	%^a^	n	%	OR (CI)	n	%	OR (CI)	n	%	OR (CI)	n	%	OR (CI)	n	%	OR (CI)	n	%	OR (CI)
**Total**	3697	58.2^b^	473	7.4		1288	20.3		373	5.9		110	1.7		107	1.7		305	4.8	
**Sex**																				
Men	2280	61.7	228	48.2	ref.	673	52.3	ref.	170	45.6	ref.	47	42.7	ref.	44	41.1	ref.	182	59.7	ref.
Women	1417	38.3	245	51.8	1.7 (1.4–2.1)	615	47.7	1.7 (1.5–1.9)	203	54.4	2.2 (1.7–2.7)	63	57.3	2.3 (1.6–3.5)	63	58.9	2.7 (1.8–4.0)	123	40.3	1.4 (1.1–1.8)
**Age group**^**c**^																				
18–40 years	2174	58.8	224	47.4	ref.	483	37.5	ref.	124	33.2	ref.	36	32.7	ref.	< 8		ref.	74	24.3	ref.
41–59 years	1523	41.2	249	52.6	1.6 (1.3–2.0)	805	62.5	1.9 (1.7–2.2)	249	66.8	2.4 (1.9–3.1)	74	67.3	2.7 (1.8–4.2)	100	93.5	22.4 (10.2–49.2)	231	75.7	5.9 (4.4–7.9)
**Level of education**^**c**^																				
Elementary	608	16.4	84	17.8	1.4 (1.1–1.9)	160	12.4	1.0 (0.8–1.3)	48	12.9	1.3 (0.9–1.8)	13	11.8	1.0 (0.5–2.0)	26	24.3	3.5 (1.9–6.4)	139	45.6	14.0 (8.9–22)
High School	1561	42.2	224	47.4	1.4 (1.1–1.7)	658	51.1	1.5 (1.3–1.8)	206	55.2	1.9 (1.5–2.5)	60	54.5	1.7 (1.1–2.6)	58	54.2	2.5 (1.5–4.1)	141	46.2	4.9 (3.2–7.6)
University/College	1528	41.3	165	34.9	ref.	470	36.5	ref.	119	31.9	ref.	37	33.6	ref.	23	21.5	ref.	25	8.2	ref.
**Country of birth**^**c**^																				
Sweden	3103	83.9	408	86.3	ref.	1104	85.7	ref.	328	87.9	ref.	92	83.6	ref.	93	86.9	ref.	254	83.3	ref.
Not Sweden	594	16.1	65	13.7	0.8 (0.6–1.1)	184	14.3	1.0 (0.8–1.2)	45	12.1	0.8 (0.5–1.1)	18	16.4	1.1 (0.7–2.0)	14	13.1	0.8 (0.4–1.4)	51	16.7	1.0 (0.7–1.4)
**Type of living area**^**c**^																				
Big cities	1388	37.5	151	31.9	ref.	438	34.0	ref.	123	33.0	ref.	36	32.7	ref.	22	20.6	ref.	71	23.3	ref.
Medium-sized cities	1553	42.0	206	43.6	1.1 (0.9–1.4)	492	38.2	1.1 (0.9–1.2)	155	41.6	1.1 (0.9–1.5)	44	40.0	1.1 (0.7–1.7)	49	45.8	1.8 (1.0–3.0)	139	45.6	1.4 (1.0–1.9)
Small cities/villages	756	20.4	116	24.5	1.3 (1.0–1.7)	358	27.8	1.4 (1.2–1.7)	95	25.5	1.3 (1.0–1.7)	30	27.3	1.4 (0.9–2.4)	36	33.6	2.5 (1.4–4.4)	95	31.1	1.6 (1.1–2.3)
**Marital status**^**c**^																				
Married	1107	29.9	157	33.2	ref.	514	39.9	ref.	143	38.3	ref.	41	37.3	ref.	41	38.3	ref.	49	16.1	ref.
Not married	2590	70.1	316	66.8	1.0 (0.8–1.2)	774	60.1	0.8 (0.7–0.9)	230	61.7	0.9 (0.7–1.1)	69	62.7	0.9 (0.6–1.4)	66	61.7	1.2 (0.8–1.9)	256	83.9	2.9 (2.1–4.1)
**Crash type**^**d**^																				
Single	3149	85.2	390	82.5	ref.	1093	84.9	ref.	315	84.5	ref.	85	77.3	ref.	90	84.1	ref.	256	83.9	ref.
Collision with pedestrian, animal, or other bicycle	204	5.5	40	8.5	1.5 (1.1–2.2)	86	6.7	1.3 (1.0–1.7)	15	4.0	0.7 (0.4–1.3)	< 8			< 8			10	3.3	0.7 (0.3–1.3)
Collision with motor vehicle	344	9.3	43	9.1	0.9 (0.7–1.3)	109	8.5	1.0 (0.8–1.3)	43	11.5	1.2 (0.8–1.7)	19	17.3	1.9 (1.1–3.2)	12	11.2	1.2 (0.6–2.2)	39	12.8	1.5 (1.0–2.2)
**Inpatient healthcare**^**d**^																				
No	3302	89.3	427	90.3	ref.	925	71.8	ref.	266	71.3	ref.	76	69.1	ref.	86	80.4	ref.	222	72.8	ref.
Yes	395	10.7	46	9.7	1.0 (0.7–1.4)	363	28.2	2.7 (2.3–3.3)	107	28.7	2.7 (2.0–3.7)	34	30.9	2.2 (1.3–3.8)	21	19.6	1.8 (1.0–3.2)	83	27.2	2.3 (1.7–3.3)
**Type of injury**^**d**^																				
Fracture	1034	28.0	91	19.2	0.5 (0.4–0.7)	789	61.3	4.3 (3.5–5.2)	200	53.6	2.6 (2.0–3.6)	34	30.9	1.3 (0.8–2.2)	42	39.3	1.2 (0.7–1.9)	126	41.3	1.6 (1.1–2.2)
Dislocation	158	4.3	18	3.8	0.7 (0.4–1.2)	78	6.1	2.8 (2.0–3.9)	16	4.3	1.6 (0.9–2.8)	< 8			< 8			< 8		
Sprains and strains	329	8.9	55	11.6	1.0 (0.7–1.4)	107	8.3	2.0 (1.5–2.7)	32	8.6	1.4 (0.9–2.2)	11	10.0	1.6 (0.8–3.3)	11	10.3	1.3 (0.6–2.6)	21	6.9	1.0 (0.6–1.7)
Internal	355	9.6	47	9.9	0.5 (0.1–2.2)	92	7.1	3.0 (1.3–6.7)	34	9.1	2.2 (0.7–6.4)	27	24.5	1.1 (0.1–8.1)	8	7.5	0.1 (0.0–3.7)	41	13.4	0.9 (0.2–3.5)
External	1728	46.7	250	52.9	ref.	207	16.1	ref.	82	22.0	ref.	35	31.8	ref.	42	39.3	ref.	103	33.8	ref.
Other and unspecified	93	2.5	12	2.5	1.2 (0.6–2.3)	15	1.2	1.9 (1.0–3.6)	9	2.4	3.3 (1.5–7.4)	< 8			< 8			< 8		
**Body region**^**d**^																				
‘Head, face, and neck, not TBI’^e^	900	24.3	94	19.9	ref.	121	9.4	ref.	30	8.0	ref.	14	12.7	ref.	17	15.9	ref.	48	15.7	ref.
‘TBI, not concussion’	23	0.6	< 8			22	1.7	2.1 (0.7–5.7)	10	2.7	4.2 (1.1–16.1)	12	10.9	18.4 (2.2–155.2)	< 8			< 8		
‘Concussion’	313	8.5	41	8.7	2.3 (0.5–10.0)	59	4.6	0.5 (0.2–1.3)	19	5.1	0.8 (0.2–2.8)	14	12.7	2.1 (0.3–17.3)	< 8			31	10.2	1.8 (0.4–7.5)
‘Spine and back’	53	1.4	11	2.3	2.7 (1.3–5.6)	43	3.3	2.3 (1.4–3.8)	15	4.0	4.5 (2.2–9.5)	< 8			< 8			< 8		
‘Torso’	261	7.1	42	8.9	1.7 (1.1–2.6)	68	5.3	1.1 (0.8–1.6)	33	8.8	2.5 (1.4–4.3)	11	10.0	2.0 (0.9–4.6)	15	14.0	2.6 (1.2–5.4)	28	9.2	1.4 (0.8–2.4)
‘Upper extremities’	1480	40.0	189	40.0	1.6 (1.2–2.2)	697	54.1	2.2 (1.7–2.8)	174	46.6	2.9 (1.9–4.5)	37	33.6	1.6 (0.8–3.2)	42	39.3	1.8 (1.0–3.4)	110	36.1	1.4 (1.0–2.1)
‘Lower extremities’	599	16.2	85	18.0	1.4 (1.0–1.9)	272	21.1	2.3 (1.7–3.0)	90	24.1	3.5 (2.2–5.5)	20	18.2	1.6 (0.8–3.4)	19	17.8	1.5 (0.7–3.0)	73	23.9	1.9 (1.2–2.9)
‘Other and unspecified’	68	1.8	< 8			< 8			< 8			< 8			< 8			< 8		

Distributions of and mutually adjusted odds ratio (OR) and 95% confidence interval (CI) for different factors in each of the seven clusters of sickness absence (SA) and disability pension (DP) status/week over 1 year before and 3 years after the date of a bicycle crash (W_−52_ to W_+156_) among 6353 individuals aged 18–59 years in December 2009 and injured in a bicycle crash in 2010, using the cluster “No SA or DP” as the reference

^a^Column percent

^b^Row percent

^c^31st December 2009

^d^At the presumed crash date

^e^Abbreviation: *TBI* Traumatic Brain Injury

**Table 4:** Distributions and crude OR for the seven cluster of SA and DP

Crude	No SA or DP		Low SA or DP			Immediate SA			Episodic SA			Long-time SA			Ongoing part-time DP			Ongoing full-time DP		
	n	%^a^	n	%	OR (CI)	n	%	OR (CI)	n	%	OR (CI)	n	%	OR (CI)	n	%	OR (CI)	n	%	OR (CI)
**Total (row%)**	3697	58.2^b^	473	7.4		1288	20.3		373	5.9		110	1.7		107	1.7		305	4.8	
**Sex**																				
Men	2280	61.7	228	48.2	ref.	673	52.3	ref.	170	45.6	ref.	47	42.7	ref.	44	41.1	ref.	182	59.7	ref.
Women	1417	38.3	245	51.8	1.7 (1.4–2.1)	615	47.7	1.5 (1.3–1.7)	203	54.4	1.9 (1.6–2.4)	63	57.3	2.2 (1.5–3.2)	63	58.9	2.3 (1.6–3.4)	123	40.3	1.1 (0.9–1.4)
**Age group, years**^**c**^																				
18–40	2174	58.8	224	47.4	ref.	483	37.5	ref.	124	33.2	ref.	36	32.7	ref.	< 8		ref.	74	24.3	ref.
41–59	1523	41.2	249	52.6	1.6 (1.3–1.9)	805	62.5	2.4 (2.1–2.7)	249	66.8	2.9 (2.3–3.6)	74	67.3	2.9 (2.0–4.4)	100	93.5	20.4 (9.5–44)	231	75.7	4.5 (3.4–5.8)
**Level of education**^**c**^																				
Elementary	608	16.4	84	17.8	1.3 (1.0–1.7)	160	12.4	0.9 (0.7–1.0)	48	12.9	1.0 (0.7–1.4)	13	11.8	0.9 (0.5–1.7)	26	24.3	2.8 (1.6–5.0)	139	45.6	14.0 (9.0–21.6)
High School	1561	42.2	224	47.4	1.3 (1.1–1.6)	658	51.1	1.4 (1.2–1.6)	206	55.2	1.7 (1.3–2.1)	60	54.5	1.6 (1.0–2.4)	58	54.2	2.5 (1.5–4.0)	141	46.2	5.5 (3.6–8.5)
University/College	1528	41.3	165	34.9	ref.	470	36.5	ref.	119	31.9	ref.	37	33.6	ref.	23	21.5	ref.	25	8.2	ref.
**Country of birth**^**c**^																				
Sweden	3103	83.9	408	86.3	ref.	1104	85.7	ref.	328	87.9	ref.	92	83.6	ref.	93	86.9	ref.	254	83.3	ref.
Not Sweden	594	16.1	65	13.7	0.8 (0.6–1.1)	184	14.3	0.9 (0.7–1.0)	45	12.1	0.7 (0.5–1.0)	18	16.4	1.0 (0.6–1.7)	14	13.1	0.8 (0.4–1.4)	51	16.7	1.0 (0.8–1.4)
**Type of living area**^**c**^																				
Big cities	1388	37.5	151	31.9	ref.	438	34.0	ref.	123	33.0	ref.	36	32.7	ref.	22	20.6	ref.	71	23.3	ref.
Medium-sized cities	1553	42.0	206	43.6	1.2 (1.0–1.5)	492	38.2	1.0 (0.9–1.2)	155	41.6	1.1 (0.9–1.4)	44	40.0	1.1 (0.7–1.7)	49	45.8	2.0 (1.2–3.3)	139	45.6	1.7 (1.3–2.3)
Small cities/villages	756	20.4	116	24.5	1.4 (1.1–1.8)	358	27.8	1.5 (1.3–1.8)	95	25.5	1.4 (1.1–1.9)	30	27.3	1.5 (0.9–2.5)	36	33.6	3.0 (1.8–5.1)	95	31.1	2.5 (1.8–3.4)
**Marital status**^**c**^																				
Married	1107	29.9	157	33.2	ref.	514	39.9	ref.	143	38.3	ref.	41	37.3	ref.	41	38.3	ref.	49	16.1	ref.
Not married	2590	70.1	316	66.8	0.9 (0.7–1.1)	774	60.1	0.6 (0.6–0.7)	230	61.7	0.7 (0.6–0.9)	69	62.7	0.7 (0.5–1.1)	66	61.7	0.7 (0.5–1.0)	256	83.9	2.2 (1.6–3.1)
**Crash type**^**d**^																				
Single	3149	85.2	390	82.5	ref.	1093	84.9	ref.	315	84.5	ref.	85	77.3	ref.	90	84.1	ref.	256	83.9	ref.
Collision with pedestrian, animal, or other bicycle	204	5.5	40	8.5	1.6 (1.1–2.3)	86	6.7	1.2 (0.9–1.6)	15	4.0	0.7 (0.4–1.3)	< 8			< 8			10	3.3	0.6 (0.3–1.2)
Collision with motor vehicle	344	9.3	43	9.1	1.0 (0.7–1.4)	109	8.5	0.9 (0.7–1.1)	43	11.5	1.2 (0.9–1.8)	19	17.3	2.0 (1.2–3.4)	12	11.2	1.2 (0.7–2.3)	39	12.8	1.4 (0.98–2.0)
**Inpatient healthcare**^**d**^																				
No	3302	89.3	427	90.3	ref.	925	71.8	ref.	266	71.3	ref.	76	69.1	ref.	86	80.4	ref.	222	72.8	ref.
Yes	395	10.7	46	9.7	0.9 (0.7–1.2)	363	28.2	3.3 (2.8–3.8)	107	28.7	3.4 (2.6–4.3)	34	30.9	3.7 (2.5–5.7)	21	19.6	2.0 (1.3–3.3)	83	27.2	3.1 (2.4–4.1)
**Type of injury**^**d**^																				
Fracture	1034	28.0	91	19.2	0.6 (0.5–0.8)	789	61.3	6.4 (5.4–7.6)	200	53.6	4.1 (3.1–5.3)	34	30.9	1.6 (1.0–2.6)	42	39.3	1.7 (1.1–2.6)	126	41.3	2.0 (1.6–2.7)
Dislocation	158	4.3	18	3.8	0.8 (0.5–1.3)	78	6.1	4.1 (3.0–5.6)	16	4.3	2.1 (1.2–3.7)	< 8			< 8			< 8		
Sprains and strains	329	8.9	55	11.6	1.2 (0.8–1.6)	107	8.3	2.7 (2.1–3.5)	32	8.6	2.0 (1.3–3.1)	11	10.0	1.7 (0.8–3.3)	11	10.3	1.4 (0.7–2.7)	21	6.9	1.1 (0.7–1.7)
Internal	355	9.6	47	9.9	0.9 (0.7–1.3)	92	7.1	2.2 (1.7–2.8)	34	9.1	2.0 (1.3–3.1)	27	24.5	3.8 (2.2–6.3)	8	7.5	0.9 (0.4–2.0)	41	13.4	1.9 (1.3–2.8)
External	1728	46.7	250	52.9	ref.	207	16.1	ref.	82	22.0	ref.	35	31.8	ref.	42	39.3	ref.	103	33.8	ref.
Other and unspecified	93	2.5	12	2.5	0.9 (0.5–1.7)	15	1.2	1.3 (0.8–2.4)	9	2.4	2.0 (1.0–4.2)	< 8			< 8			< 8		
**Body region**^**d**^																				
‘Head, face and neck, not TBI’^e^	900	24.3	94	19.9	ref.	121	9.4	ref.	30	8.0	ref.	14	12.7	ref.	17	15.9	ref.	48	15.7	ref.
‘TBI. not concussion’	23	0.6	< 8			22	1.7	7.1 (3.8–13.2)	10	2.7	13 (5.7–29.8)	12	10.9	33.5 (14–80.5)	< 8			< 8		
‘Concussion’	313	8.5	41	8.7	1.3 (0.9–1.9)	59	4.6	1.4 (1.0–2.0)	19	5.1	1.8 (1.0–3.3)	14	12.7	2.9 (1.4–6.1)	< 8			31	10.2	1.9 (1.2–3.0)
‘Spine and back’	53	1.4	11	2.3	2.0 (1.0–3.9)	43	3.3	6.0 (3.9–9.4)	15	4.0	8.5 (4.3–16.7)	< 8			< 8			< 8		
‘Torso’	261	7.1	42	8.9	1.5 (1.0–2.3)	68	5.3	1.9 (1.4–2.7)	33	8.8	3.8 (2.3–6.3)	11	10.0	2.7 (1.2–6.0)	15	14.0	3.0 (1.5–6.2)	28	9.2	2.0 (1.2–3.3)
‘Upper extremities’	1480	40.0	189	40	1.2 (0.9–1.6)	697	54.1	3.5 (2.8–4.3)	174	46.6	3.5 (2.4–5.2)	37	33.6	1.6 (0.9–3.0)	42	39.3	1.5 (0.9–2.7)	110	36.1	1.4 (1.0–2.0)
‘Lower extremities’	599	16.2	85	18	1.4 (1.0–1.9)	272	21.1	3.4 (2.7–4.3)	90	24.1	4.5 (2.9–6.9)	20	18.2	2.1 (1.1–4.3)	19	17.8	1.7 (0.9–3.3)	73	23.9	2.3 (1.6–3.3)
‘Other and unspecified’	68	1.8	< 8			< 8			< 8			< 8			< 8			< 8		

Distributions and crude odds ratio (OR) and 95% confidence interval (CI) for different factors in each of the seven cluster of sickness absence (SA) and disability pension (DP) status/week over one year before and three years after date of a bicycle crash (W_−52_ to W_+156_) among 6353 individuals aged 18–59 years in December 2009 and injured in a bicycle crash in 2010, using the cluster “No SA or DP” as the reference

^a^Column percent

^b^Row percent

^c^31st December 2009

^d^At the presumed crash date

^e^Abbreviation: *TBI* Traumatic Brain Injury

The individuals in the cluster “Low SA or DP” (7.4% of the individuals) had little SA or DP during follow-up. Further, no increase of individuals with SA or DP were observed in connection to the presumed crash date in this cluster (Fig. 2).

The cluster “Immediate SA” (20.3% of the cohort) was characterised by SA directly in relation to the crash, almost no SA before, and already within 1 year almost no one had SA. This cluster was associated with a higher proportion of individuals with fractures (OR 4.3; 95% CI 3.5–5.2) compared to external injuries. External injuries had its smallest proportion in this cluster, 16.1%, to be compared with 52.9% in the cluster “Low SA or DP”. Moreover, the injuries to the ‘spine and back’ (OR 2.3; 1.4–3.8), ‘upper extremities’ (OR 2.2; 1.7–2.8) and ‘lower extremities’ (OR 2.3; 1.7–3.0) compared with ‘head, face, and neck, not TBI’ were associated with this cluster.

In the cluster “Episodic SA” (5.9% of the cohort) most of the individuals started a new SA spell in W_0_ and then had one or several new SA spells during the follow-up. This cluster was associated with higher ORs of fractures (OR 2.6; 2.0–3.6) compared with external injuries and higher proportions of ‘TBI, not concussion’ (OR 4.2; 1.1–16.1), injuries to the ‘spine and back’ (OR 4.5; 2.2–9.5), ‘torso’ (OR 2.5; 1.4–4.3), ‘upper extremities’ (OR 2.9; 1.9–4.5), and ‘lower extremities’ (OR 3.5; 2.2–5.5) compared with injuries to the ‘head, face, and neck, not TBI’.

In the cluster “Long-term SA” (1.7% of the cohort), the individuals were on SA during almost all of the follow-up time. About one third of them were also on SA or DP during the year before the crash, this rate increased to about two thirds at W_0_ and almost everyone in this cluster were on SA or DP 2 years after W_0_. This cluster was associated with higher proportion of the included individuals being in a collision with a motor vehicle (OR1.9; 1.1–3.2) and ‘TBI, not concussion’ (OR 18.4; 2.2–155.2). The proportions of ‘TBI, not concussion’ in this cluster was 10.9%, to be compared with the proportions ranging between 0.6 and 2.7% in the other clusters.

The individuals in the cluster “Ongoing part-time DP” (1.7% of the cohort) had part-time DP or combined SA and DP during the follow-up, with more individuals with combined SA and DP at W_0_.

The individuals in the cluster “Ongoing full-time DP” (4.8% of the cohort), were on full-time DP through almost all of the four studied years. This cluster was associated with a higher proportion of not married individuals compared to in the cluster “no SA or DP” (OR 2.9; 2.1–4.1).

**Fig. 3:**
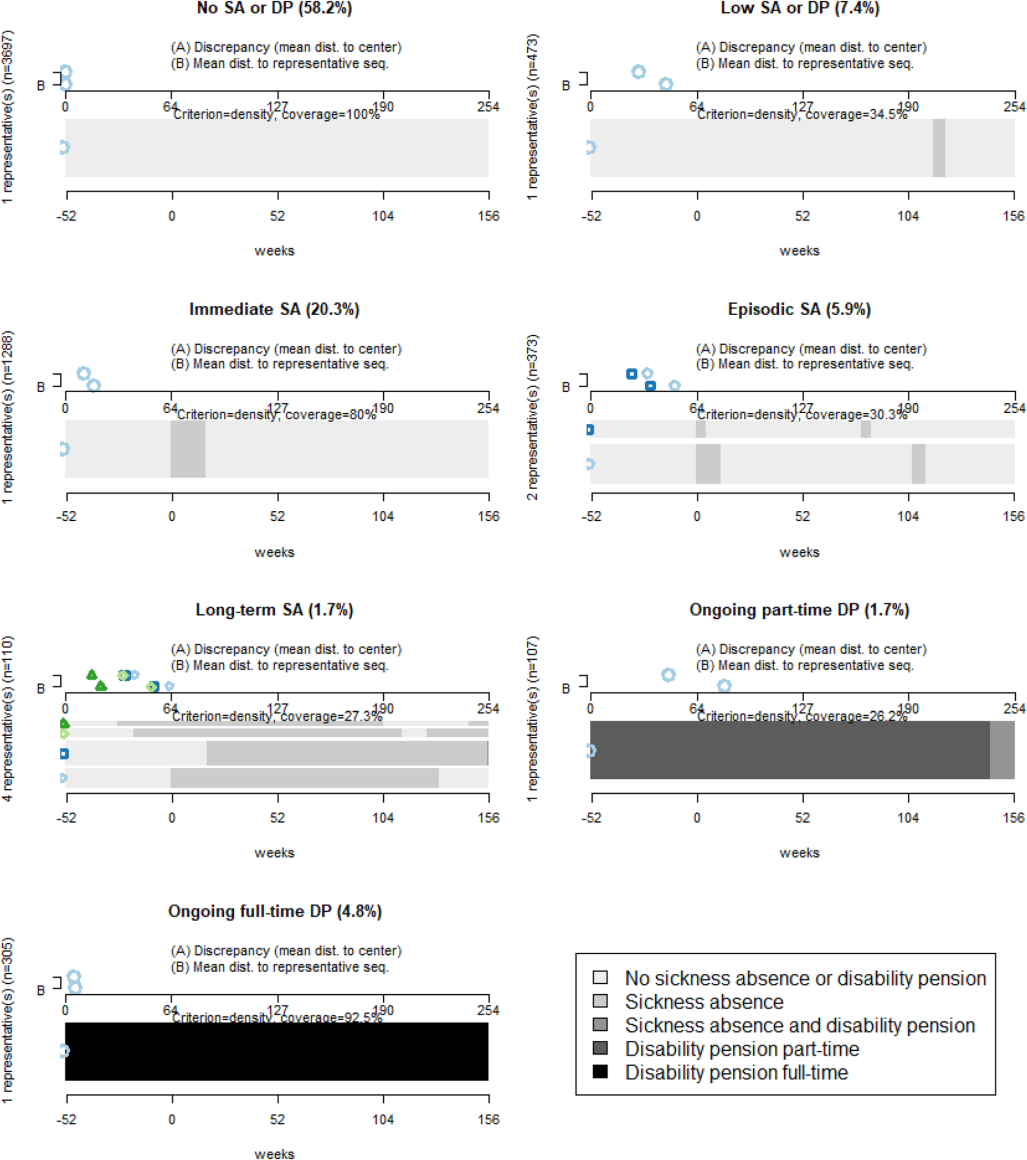
Representative sequence(s) for the seven identified clusters. Representative sequence(s) that with a neighbourhood radius of 10% cover(s) at least 25% of all sequences in each cluster of SA and/or DP states/week during one year before up until three years after (W_−52_ to W_+156_) the week of the bicycle crash (marked with 0 in the figure), for the seven identified clusters. The proportion of the study population (*N* = 6353) is stated in each cluster heading. Discrepancy (A) and mean distances (B) to each representative sequence are presented, marked with different symbols for each representative sequence

To summarize the sequences of the different clusters and to see the heterogeneity or homogeneity in each cluster, a set of representative sequences for each cluster were extracted. The representative sequence(s) for each of the seven clusters are shown in Fig. 3. The clusters “No SA or DP” and “Ongoing full-time DP” are homogeneous clusters, where the representative sequence covers 100 and 92.5% of the sequences in their clusters respectively. For the clusters “Episodic SA” and “Long-term SA”, there was more heterogeneity among the representative sequences. “Episodic SA” had two different representative sequences covering together 30.3% of the sequences in that cluster. Both representative sequences showed a SA spell starting at W_0,_ but of different lengths and both representative sequences had one additional SA spell during the follow-up but located at different time points. In the cluster “Long-term SA” there were four representative sequences covering 27.3% of the sequences in that cluster, showing an even more heterogenic cluster. These representative sequences showed long periods of SA but with different starting points in relation to the crash, one of the representative sequences started SA at W_0_, two of the representative sequences started SA before W_0_, and one representative sequence started SA after W_0_.

In the sensitivity analyses, also including those 112 who died or emigrated during the follow-up, the result did not change substantially.

## Discussion

In this nationwide longitudinal cohort study, investigating SA and DP during 4 years among all 6353 individuals of working age who in 2010 had a new bicycle crash leading to in-patient or specialized out-patient healthcare and were alive and living in Sweden during the follow-up, we identified seven different clusters of SA and DP sequences. The sequences display the diversity in SA and DP, both before and after a bicycle crash. The seven clusters were named: “No SA or DP” (58.2% of all in the cohort), “Low SA or DP” (7.4%), “Immediate SA” (20.3%), “Episodic SA” (5.9%), “Long-term SA” (1.7%), “Ongoing part-time DP” (1.7%), and “Ongoing full-time DP” (4.8%). Compared with the cluster “No SA or DP”, all other clusters were associated with higher proportions of women, individuals in the highest age group, and individuals with high school education (compared with university/college). All clusters, except “Low SA and DP”, had high ORs for in-patient healthcare at the presumed crash date. The clusters “Immediate SA” and “Episodic SA” were associated with high proportions of fractures and the cluster “Long-term SA” was associated with a high proportion of ‘TBI, not concussion’ 10.9% compared with proportions, ranging between 0.6 and 2.7%, in the other clusters. To the best of our knowledge, the present study is the first nationwide register-based study exploring the heterogeneity of SA and DP in individuals injured in a bicycle crash using sequence analysis.

In the present study we found that 66% had no or little SA and DP during the three follow-up years. This is in line with a Swedish study using group-based trajectory models, where 76% of the included 903 individuals who had a road traffic injury were found in a pattern with a low number of SA days during the three-year follow-up [10]. However, that study did not consider DP during the follow-up nor were different road user groups specified. Our study showed a variety of SA and DP in the seven different clusters, and especially that previous SA was common in the clusters with more SA in the years following the bicycle crash. DP was mostly observed in two separate clusters. To obtain a complete picture of individuals with reduced work capacity due to disease or injury, both SA and DP need to be taken into consideration otherwise the total number of days lost will be underestimated. Moreover, SA may lead to DP and those on full-time DP are not at risk of SA. Further, all SA and DP irrespective of diagnoses are important to take into consideration as an injury can lead to various SA/DP diagnoses besides the initial injury diagnosis. In addition, the crash might worsen the symptoms of an underlying disease, prolonging an already ongoing SA or worsen it so that it leads to the need of new SA. To investigate this further, previous morbidity or SA diagnoses should be taken into consideration in future studies.

Our study showed an association between having been hospitalized and being in the clusters “Immediate SA”, “Episodic SA”, “Long-term SA”, “Ongoing part-time DP”, or “Ongoing full-time DP”. The association between SA and hospitalisation was also observed in two previous old and relatively small studies [12, 13]. Likewise, the association between age and SA and/or DP is consistent with previous studies reporting on this association [7, 10, 13].

In a study from Finland of 264 individuals injured in a bicycle crash in 1985–1986 it was found that those, among the hospitalized individuals, injured in a collision with a motor vehicle had longer self-reported disability to work than those hospitalized for other bicycle injuries [13]. This may be due to greater crash severity in such collisions. In our study, this was observed in the cluster “Long-term SA”, but not in the other clusters, where other factors were of more importance.

For the clusters “No SA or DP” and “Low SA or DP”, the most common type of injuries were external injuries and most of the injuries were located to the ‘head, face, and neck, not TBI’, and to the ‘upper extremities’. These injuries are minor and hence are not expected to lead to SA and DP to an equal extent as the other injures. In another study, individuals with wound injuries or contusions were also shown to have less time on SA or DP. Individuals with fractures, luxation, and distortions had a very long mean period on SA or DP [12]. This could also be seen in our clusters “Immediate SA” and “Episodic SA”, whereas in the cluster “Long-term SA” ‘TBI, not concussion’, had the highest OR for being in that cluster (OR: 18.4 CI: 2.2–155.2). We have previously shown that those with TBI and spinal injuries have high OR for SA ≥90 days in a Swedish cross-sectional study of bicycle crashes 2009–2011 using the same data sources as in this study [14]. A Finnish study of 264 adults injured in a bicycle crash observed that upper extremity injuries was the most frequent reason for self-reported disability to work [13]. This is in line with our results regarding that the three clusters “Low SA or DP”, “Immediate SA”, and “Episodic SA” were associated with a higher proportion of injuries to the ‘upper extremities’.

Using sequence and cluster analysis provided a more comprehensive picture of various SA and DP patterns among injured bicyclist. This can be seen as a good complement to traditional regression analysis [30–32, 43]. The advantage of methods allowing for complex pattern analysis is illustrated in the present study especially by the clusters “Episodic SA” and “Long-term SA”, where the heterogeneity of SA and DP can be seen. The heterogeneity in the cluster “Long-term SA” could be due to this cluster consisting of very different individuals, not only those with long-term SA due to bicycle crash, but also those with long-term SA starting before the crash, and long-term SA arising later during follow-up. The later could both be due to late effect of the bicycle crash but also due to other diagnoses. In addition, the knowledge of this heterogeneity in SA and DP could be of great importance for authorities, policy makers, insurance companies, healthcare and individual’s rehabilitation plans, and plans regarding future work. However, more studies are needed. Some aspects that could be taken into consideration in future research are, e.g., to obtain information also on visits to primary healthcare, to study different SA and DP diagnoses, to compare the results with matched references from the general population or other injured individuals, and to compare the results from different years to see trends or effect of injury prevention.

### Strengths and limitations

Strengths of the present study are that data from high-quality nationwide registers were used, with total population coverage of all residents in Sweden of working age, that all diagnoses were register-based (thus, certified by a physician) rather than self-reported, no drop-outs, and the very large study population, allowing for more clusters and categorisations. The register data also allowed for several years of complete follow-up information. Furthermore, a strength with sequence analysis is that it takes advantage of the rich data to make a full description of the complex SA and DP patterns. This study covers all individuals receiving in-patient and/or specialized out-patient healthcare, i.e., all bicycle crashes severe enough to acquire such medical attention were included. According to national statistics 21 bicyclists were fatally injured and 1760 were severely injured in 2010 in Sweden. By using in-patient and specialized out-patient register for individuals of working ages, more bicycle crashes than the bicycle crashes registered in the national system for road traffic injury data collection in Sweden will be captured. In assessments of the possible negative consequences of bicycling, previous studies have mainly referred to fatalities or police-reported crashes [44, 45]. That will not adequately describe the situation, e.g., in Sweden the police reports only cover around 7% of all bicycle crashes [46]. This under-reporting of the number of crashes to the police has also been shown in other countries [47]. Healthcare data covers a much larger proportion of bicycle crashes and the use of such data is, therefore, a strength when studying individuals injured in bicycle crashes [46, 48]. A limitation in using this type of data is that the actual date of the crash is not always known, just the first date of the visit/hospitalization, nor is some information available about the crash, such as helmet use, time of the day, type of environment, etc. Another limitation is that the selection of only one injury diagnosis might have led to over- or under-estimation of the impact of different diagnoses. However, the majority (78%) had only one injury diagnosis registered. As we excluded all with a previous healthcare visit due to a traffic crash (in order only to get visits due to a new crash) we might have missed people with several crashes. It can also be considered a limitation that individuals with a bicycle injury not requiring healthcare or only requiring primary healthcare were not included; that is, our results will underestimate the total number of bicycle crashes, primarily the less severe injuries. Another limitation is that we only have information on SA spells > 14 days, this is important since most SA spells are shorter than this. However, the longer spells contribute with the most number of SA days in total [49, 50].

## Conclusions

Seven clusters with different patterns of SA and DP were identified, showing the complexity of SA and DP before and after a bicycle crash. There were two clusters with no or very little SA and DP during the follow-up, three clusters with different levels of SA, and two clusters with different extent of DP. The majority did not have any SA or DP in relation to the bicycle crash. Fractures were more frequent in the clusters “Immediate SA” and “Episodic SA”. Whereas ‘TBI, not concussion’ and individuals injured in collision with motor vehicle were more frequent in the cluster “Long-term SA”. This knowledge could be used for targeted interventions for reducing SA and DP after a bicycle crash, with focus on injuries with a high proportion of long-term SA or DP rather than the most common injuries.

## Data Availability

The data cannot be made publically available, according to privacy regulations. According to the General Data Protection Regulation, the Swedish law SFS 2018:218, the Swedish Data Protection Act, the Swedish Ethical Review Act, and the Public Access to Information and Secrecy Act, data can only be made available, after legal review, for researchers who meet the criteria for access to this type of sensitive and confidential data. Readers may contact professor Kristina Alexanderson (kristina.alexanderson@ki.se) regarding the data.

## Abbreviations

CI: Confidence Interval
DP: Disability Pension
EU: European Union
ICD-10: International Classification of Diseases, version 10
OR: Odds Ratio
SA: Sickness Absence
TBI: Traumatic Brain Injury
W_0_: Week zero, the week of three days before and three days after the presumed crash date
